# *N*-(1-Naphth­yl)acetoacetamide

**DOI:** 10.1107/S1600536808002638

**Published:** 2008-01-30

**Authors:** Xi-Shi Tai, Yi-Min Feng, Hua-Xiang Zhang

**Affiliations:** a Department of Chemistry, Weifang University, Weifang 261061, People’s Republic of China

## Abstract

The title compound, C_14_H_13_NO_2_, exists in the keto form. An N—H⋯O hydrogen bond helps to establish the packing.

## Related literature

For background, see: Huang *et al.* (2001 ▶).
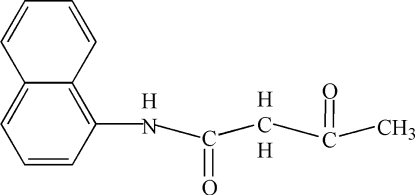


## Experimental

### 

#### Crystal data

C_14_H_13_NO_2_
*M* *_r_* = 227.25
Monoclinic, 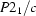

*a* = 17.856 (2) Å
*b* = 8.1076 (12) Å
*c* = 8.5153 (14) Å
β = 102.777 (2)°
*V* = 1202.2 (3) Å^3^
*Z* = 4
Mo *K*α radiation
μ = 0.08 mm^−1^
*T* = 298 (2) K
0.50 × 0.40 × 0.38 mm

#### Data collection

Bruker SMART CCD diffractometer
Absorption correction: multi-scan (*SADABS*; Bruker, 2000 ▶) *T*_min_ = 0.959, *T*_max_ = 0.969
5815 measured reflections
2116 independent reflections
1335 reflections with *I* > 2σ(*I*)
*R*_int_ = 0.042

#### Refinement

*R*[*F* ^2^ > 2σ(*F* ^2^)] = 0.043
*wR*(*F* ^2^) = 0.121
*S* = 1.05
2116 reflections
155 parameters
H-atom parameters constrained
Δρ_max_ = 0.15 e Å^−3^
Δρ_min_ = −0.17 e Å^−3^

### 

Data collection: *SMART* (Bruker, 2000 ▶); cell refinement: *SAINT* (Bruker, 2000 ▶); data reduction: *SAINT*; program(s) used to solve structure: *SHELXS97* (Sheldrick, 2008 ▶); program(s) used to refine structure: *SHELXL97* (Sheldrick, 2008 ▶); molecular graphics: *SHELXTL* (Sheldrick, 2008 ▶); software used to prepare material for publication: *SHELXTL*.

## Supplementary Material

Crystal structure: contains datablocks global, I. DOI: 10.1107/S1600536808002638/hb2694sup1.cif

Structure factors: contains datablocks I. DOI: 10.1107/S1600536808002638/hb2694Isup2.hkl

Additional supplementary materials:  crystallographic information; 3D view; checkCIF report

## Figures and Tables

**Table 1:** Hydrogen-bond geometry (Å, °)

*D*—H⋯*A*	*D*—H	H⋯*A*	*D*⋯*A*	*D*—H⋯*A*
N1—H1⋯O1^i^	0.86	2.01	2.853 (2)	168

Symmetry code: (i) 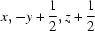.

**Fig. 1.**
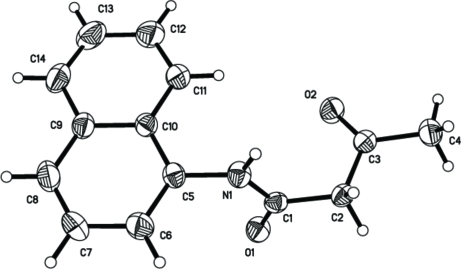
The molecular structure of (I) showing 30% probabiility ellipsoids (arbitrary spheres for the H atoms).

## supplementary crystallographic information

### Comment

The europium(III) and terbium(III) complexes of beta-diketonato and related conjugated ligands have been studied as emitting materials for organic light emitting diodes (OLEDs) (*e.g.* Huang *et al.*, 2001). However, the quantum efficiency of most these complexes are unfortunately still low. This may be due to inefficiency of the trpilet-triplet energy transfer in these complexes. Therefore, there is a need to design ligands which have better energy transfer properties when coordinated to the lanthanide metal ion. As part of our studies in this area, we now report the synthesis and structure of the title compound, (I).

In (I), the C=O bonds length are 1.226 (2)Å and 1.208 (2) Å, indicating that it exists in the keto form (Fig. 1) in the solid state.

In the crystal structure, the molecules are stabilized by an N—H···O intermolecular hydrogen bond (Table 1) leading to [001] chains.

### Experimental

A solution of 1-naphthaline (10 mmol) in 30 ml benzene was added to a solution of ethyl acetoacetate (10 mmol). The reaction mixture was refluxed for 2 h with stirring, then the resulting pale precipitate was obtained by filtration, washed several times with benzene and dried *in vacuo* (yield 89%). Colourless blocks of (I) were recrystallized from ethanol by slow evaporation. IR (KBr, cm^-1^): 3242 (m, N—H), 1723 (s, CH_3_C=O), 1665 (s, amide C=O).

### Refinement

The H atoms were geometrically placed (C—H = 0.93–0.97 Å, N—H = 0.86 Å) and refined as riding with *U*_iso_(H) = 1.2*U*_eq_(C, N) or 1.5*U*_eq_(methyl C).

### Crystal data

**Table:**

C_14_H_13_NO_2_	*F*_000_ = 480
*M**_r_* = 227.25	*D*_x_ = 1.256 Mg m^−^^3^
Monoclinic, *P*2_1_/*c*	Mo *K*α radiation λ = 0.71073 Å
Hall symbol: -P 2ybc	Cell parameters from 1643 reflections
*a* = 17.856 (2) Å	θ = 2.3–23.5º
*b* = 8.1076 (12) Å	µ = 0.08 mm^−^^1^
*c* = 8.5153 (14) Å	*T* = 298 (2) K
β = 102.777 (2)º	Block, colourless
*V* = 1202.2 (3) Å^3^	0.50 × 0.40 × 0.38 mm
*Z* = 4	

### Data collection

**Table:**

Bruker SMART CCD diffractometer	2116 independent reflections
Radiation source: fine-focus sealed tube	1335 reflections with *I* > 2σ(*I*)
Monochromator: graphite	*R*_int_ = 0.042
*T* = 298(2) K	θ_max_ = 25.0º
ω scans	θ_min_ = 2.3º
Absorption correction: multi-scan(SADABS; Bruker, 2000)	*h* = −21→21
*T*_min_ = 0.959, *T*_max_ = 0.969	*k* = −9→6
5815 measured reflections	*l* = −10→10

### Refinement

**Table:**

Refinement on *F*^2^	Secondary atom site location: difference Fourier map
Least-squares matrix: full	Hydrogen site location: inferred from neighbouring sites
*R*[*F*^2^ > 2σ(*F*^2^)] = 0.043	H-atom parameters constrained
*wR*(*F*^2^) = 0.121	*w* = 1/[σ^2^(*F*_o_^2^) + (0.0402*P*)^2^ + 0.3082*P*] where *P* = (*F*_o_^2^ + 2*F*_c_^2^)/3
*S* = 1.05	(Δ/σ)_max_ < 0.001
2116 reflections	Δρ_max_ = 0.15 e Å^−^^3^
155 parameters	Δρ_min_ = −0.17 e Å^−^^3^
Primary atom site location: structure-invariant direct methods	Extinction correction: none

### Special details

**Table:**

Geometry. All e.s.d.'s (except the e.s.d. in the dihedral angle between two l.s. planes) are estimated using the full covariance matrix. The cell e.s.d.'s are taken into account individually in the estimation of e.s.d.'s in distances, angles and torsion angles; correlations between e.s.d.'s in cell parameters are only used when they are defined by crystal symmetry. An approximate (isotropic) treatment of cell e.s.d.'s is used for estimating e.s.d.'s involving l.s. planes.
Refinement. Refinement of *F*^2^ against ALL reflections. The weighted *R*-factor *wR* and goodness of fit *S* are based on *F*^2^, conventional *R*-factors *R* are based on *F*, with *F* set to zero for negative *F*^2^. The threshold expression of *F*^2^ > σ(*F*^2^) is used only for calculating *R*-factors(gt) *etc*. and is not relevant to the choice of reflections for refinement. *R*-factors based on *F*^2^ are statistically about twice as large as those based on *F*, and *R*- factors based on ALL data will be even larger.

### Fractional atomic coordinates and isotropic or equivalent isotropic displacement parameters (Å^2^)

**Table:**

	*x*	*y*	*z*	*U*_iso_*/*U*_eq_	
N1	0.21814 (9)	0.2430 (2)	0.4659 (2)	0.0487 (5)	
H1	0.2099	0.2073	0.5557	0.058*	
O1	0.16833 (8)	0.38359 (18)	0.23841 (18)	0.0561 (4)	
O2	0.05169 (8)	0.08943 (19)	0.29965 (19)	0.0631 (5)	
C1	0.16240 (11)	0.3270 (2)	0.3687 (3)	0.0429 (5)	
C2	0.08902 (11)	0.3457 (2)	0.4262 (2)	0.0445 (5)	
H2A	0.1011	0.3525	0.5428	0.053*	
H2B	0.0642	0.4480	0.3845	0.053*	
C3	0.03443 (11)	0.2049 (3)	0.3741 (2)	0.0453 (5)	
C4	−0.04209 (12)	0.2179 (3)	0.4158 (3)	0.0648 (7)	
H4A	−0.0724	0.1229	0.3757	0.097*	
H4B	−0.0677	0.3157	0.3679	0.097*	
H4C	−0.0355	0.2236	0.5307	0.097*	
C5	0.29076 (11)	0.2094 (3)	0.4290 (2)	0.0451 (5)	
C6	0.34536 (13)	0.3281 (3)	0.4510 (3)	0.0623 (6)	
H6	0.3347	0.4324	0.4860	0.075*	
C7	0.41772 (14)	0.2943 (4)	0.4212 (3)	0.0737 (8)	
H7	0.4547	0.3769	0.4355	0.088*	
C8	0.43444 (13)	0.1437 (3)	0.3720 (3)	0.0694 (7)	
H8	0.4834	0.1227	0.3557	0.083*	
C9	0.37919 (12)	0.0178 (3)	0.3450 (3)	0.0531 (6)	
C10	0.30501 (11)	0.0509 (3)	0.3728 (2)	0.0460 (5)	
C11	0.24937 (13)	−0.0750 (3)	0.3404 (3)	0.0631 (7)	
H11	0.2006	−0.0558	0.3583	0.076*	
C12	0.26597 (16)	−0.2243 (3)	0.2834 (4)	0.0827 (9)	
H12	0.2284	−0.3057	0.2616	0.099*	
C13	0.33885 (17)	−0.2560 (4)	0.2574 (4)	0.0852 (9)	
H13	0.3496	−0.3584	0.2186	0.102*	
C14	0.39381 (15)	−0.1396 (3)	0.2880 (3)	0.0719 (7)	
H14	0.4424	−0.1634	0.2713	0.086*	

### Atomic displacement parameters (Å^2^)

**Table:**

	*U*^11^	*U*^22^	*U*^33^	*U*^12^	*U*^13^	*U*^23^
N1	0.0492 (10)	0.0576 (11)	0.0413 (10)	0.0042 (9)	0.0144 (8)	0.0041 (8)
O1	0.0621 (9)	0.0623 (10)	0.0472 (9)	0.0061 (7)	0.0196 (7)	0.0069 (8)
O2	0.0646 (10)	0.0575 (10)	0.0711 (11)	−0.0038 (8)	0.0237 (8)	−0.0201 (8)
C1	0.0510 (12)	0.0366 (11)	0.0421 (12)	−0.0014 (9)	0.0124 (10)	−0.0047 (10)
C2	0.0494 (12)	0.0423 (12)	0.0427 (12)	0.0050 (9)	0.0124 (9)	−0.0022 (9)
C3	0.0513 (12)	0.0463 (13)	0.0388 (12)	0.0039 (10)	0.0110 (10)	0.0022 (10)
C4	0.0586 (14)	0.0655 (16)	0.0763 (17)	−0.0046 (12)	0.0281 (13)	−0.0085 (13)
C5	0.0398 (11)	0.0541 (13)	0.0406 (12)	−0.0009 (10)	0.0072 (9)	0.0017 (10)
C6	0.0578 (14)	0.0590 (15)	0.0686 (16)	−0.0085 (12)	0.0109 (12)	−0.0098 (12)
C7	0.0507 (15)	0.0733 (19)	0.096 (2)	−0.0191 (13)	0.0133 (14)	−0.0021 (16)
C8	0.0432 (13)	0.084 (2)	0.0812 (18)	−0.0005 (13)	0.0156 (12)	0.0075 (15)
C9	0.0444 (12)	0.0598 (15)	0.0547 (14)	0.0070 (11)	0.0101 (10)	0.0058 (11)
C10	0.0407 (11)	0.0494 (13)	0.0459 (13)	0.0014 (10)	0.0053 (9)	0.0045 (10)
C11	0.0501 (13)	0.0556 (15)	0.0823 (18)	−0.0022 (11)	0.0115 (12)	−0.0008 (13)
C12	0.0718 (18)	0.0572 (17)	0.114 (2)	−0.0058 (14)	0.0102 (16)	−0.0108 (16)
C13	0.091 (2)	0.0591 (17)	0.103 (2)	0.0171 (16)	0.0165 (18)	−0.0126 (16)
C14	0.0645 (16)	0.0731 (18)	0.0809 (19)	0.0219 (14)	0.0223 (14)	0.0032 (15)

### Geometric parameters (Å, °)

**Table:**

N1—C1	1.332 (2)	C6—H6	0.9300
N1—C5	1.427 (2)	C7—C8	1.346 (3)
N1—H1	0.8600	C7—H7	0.9300
O1—C1	1.226 (2)	C8—C9	1.403 (3)
O2—C3	1.208 (2)	C8—H8	0.9300
C1—C2	1.504 (3)	C9—C14	1.410 (3)
C2—C3	1.503 (3)	C9—C10	1.422 (3)
C2—H2A	0.9700	C10—C11	1.408 (3)
C2—H2B	0.9700	C11—C12	1.361 (3)
C3—C4	1.490 (3)	C11—H11	0.9300
C4—H4A	0.9600	C12—C13	1.391 (4)
C4—H4B	0.9600	C12—H12	0.9300
C4—H4C	0.9600	C13—C14	1.344 (4)
C5—C6	1.353 (3)	C13—H13	0.9300
C5—C10	1.413 (3)	C14—H14	0.9300
C6—C7	1.398 (3)		
			
C1—N1—C5	123.47 (17)	C7—C6—H6	119.9
C1—N1—H1	118.3	C8—C7—C6	120.8 (2)
C5—N1—H1	118.3	C8—C7—H7	119.6
O1—C1—N1	123.44 (18)	C6—C7—H7	119.6
O1—C1—C2	120.82 (19)	C7—C8—C9	121.0 (2)
N1—C1—C2	115.72 (18)	C7—C8—H8	119.5
C3—C2—C1	112.45 (16)	C9—C8—H8	119.5
C3—C2—H2A	109.1	C8—C9—C14	122.5 (2)
C1—C2—H2A	109.1	C8—C9—C10	118.9 (2)
C3—C2—H2B	109.1	C14—C9—C10	118.6 (2)
C1—C2—H2B	109.1	C11—C10—C5	123.57 (19)
H2A—C2—H2B	107.8	C11—C10—C9	118.3 (2)
O2—C3—C4	122.28 (19)	C5—C10—C9	118.16 (18)
O2—C3—C2	121.29 (18)	C12—C11—C10	121.0 (2)
C4—C3—C2	116.41 (18)	C12—C11—H11	119.5
C3—C4—H4A	109.5	C10—C11—H11	119.5
C3—C4—H4B	109.5	C11—C12—C13	120.4 (2)
H4A—C4—H4B	109.5	C11—C12—H12	119.8
C3—C4—H4C	109.5	C13—C12—H12	119.8
H4A—C4—H4C	109.5	C14—C13—C12	120.5 (3)
H4B—C4—H4C	109.5	C14—C13—H13	119.7
C6—C5—C10	121.01 (19)	C12—C13—H13	119.7
C6—C5—N1	119.7 (2)	C13—C14—C9	121.3 (2)
C10—C5—N1	119.28 (17)	C13—C14—H14	119.4
C5—C6—C7	120.1 (2)	C9—C14—H14	119.4
C5—C6—H6	119.9		
			
C5—N1—C1—O1	−0.2 (3)	N1—C5—C10—C11	4.3 (3)
C5—N1—C1—C2	178.25 (17)	C6—C5—C10—C9	2.0 (3)
O1—C1—C2—C3	90.1 (2)	N1—C5—C10—C9	−176.73 (18)
N1—C1—C2—C3	−88.3 (2)	C8—C9—C10—C11	178.1 (2)
C1—C2—C3—O2	2.6 (3)	C14—C9—C10—C11	−0.6 (3)
C1—C2—C3—C4	−175.97 (18)	C8—C9—C10—C5	−0.9 (3)
C1—N1—C5—C6	79.3 (3)	C14—C9—C10—C5	−179.6 (2)
C1—N1—C5—C10	−101.9 (2)	C5—C10—C11—C12	178.7 (2)
C10—C5—C6—C7	−1.3 (3)	C9—C10—C11—C12	−0.3 (3)
N1—C5—C6—C7	177.5 (2)	C10—C11—C12—C13	0.7 (4)
C5—C6—C7—C8	−0.7 (4)	C11—C12—C13—C14	−0.1 (5)
C6—C7—C8—C9	1.8 (4)	C12—C13—C14—C9	−0.8 (4)
C7—C8—C9—C14	177.7 (2)	C8—C9—C14—C13	−177.4 (3)
C7—C8—C9—C10	−0.9 (4)	C10—C9—C14—C13	1.2 (4)
C6—C5—C10—C11	−176.9 (2)		

### Hydrogen-bond geometry (Å, °)

**Table:**

*D*—H···*A*	*D*—H	H···*A*	*D*···*A*	*D*—H···*A*
N1—H1···O1^i^	0.86	2.01	2.853 (2)	168

Symmetry codes: (i) *x*, −*y*+1/2, *z*+1/2.
